# Single-cell transcriptomics of acetaminophen-induced responses in human 2D and 3D liver microtissues

**DOI:** 10.1007/s00204-025-04296-6

**Published:** 2026-01-14

**Authors:** Brian Bwanya, Marcha C. T. Verheijen, Duncan Hauser, Theo M. de Kok, Danyel G. J. Jennen, Twan van den Beucken, Florian Caiment

**Affiliations:** Department of Translational Genomics, GROW Research Institute for Oncology and Developmental Biology, Maastricht University, 6229 ER Maastricht, The Netherlands

**Keywords:** Single-cell transcriptomics, Acetaminophen toxicity, Drug metabolism, Hypoxia, DILI

## Abstract

Drug-induced liver injury remains a major obstacle in pharmaceutical development and a leading cause of acute liver failure, underscoring the need for predictive and human-relevant in vitro models. Acetaminophen, a widely used analgesic with well-characterized dose-dependent hepatotoxicity, serves as a benchmark compound for evaluating liver toxicity mechanisms. Here, we applied single cell RNA sequencing to characterize cellular responses to acetaminophen exposure in two distinct liver cell culture formats: two-dimensional monolayers (2D) and three-dimensional (3D) spheroids composed of primary human hepatocytes, Kupffer cells, and liver endothelial cells. Cultures were exposed for 24 h to low (350 µM) and high (2687 µM) acetaminophen concentrations, with 2D cultures receiving only the low dose. Compared to 2D monolayers, 3D spheroids exhibited greater transcriptional diversity and elevated expression of ribosomal genes, indicative of enhanced metabolic and biosynthetic activity. Reactome pathway analysis revealed pronounced hypoxia-associated signaling in 3D cultures even under baseline conditions, likely due to restricted oxygen diffusion within spheroids. These hypoxia signatures were most prominent in endothelial cells. Upon acetaminophen exposure, hypoxic hepatocytes displayed elevated expression of cytochrome P450 enzymes, while conjugation enzymes involved in detoxification declined with increasing dose, suggesting compromised phase II metabolism under oxygen-limited conditions. Normoxic hepatocytes showed minimal transcriptional response. These results suggest a dynamic interplay between oxygen availability and acetaminophen metabolism, where oxygen tension influences metabolic activity, and drug metabolism in turn alters the hypoxic landscape. Our findings underscore the physiological relevance of 3D liver models and highlight the importance of spatial microenvironmental context for improving mechanistic insights into hepatotoxicity.

**Supplementary Information:**

The online version contains supplementary material available at 10.1007/s00204-025-04296-6.

## Introduction

Acetaminophen (APAP) is extensively studied in drug-induced liver injury (DILI), with studies spanning animal models and human overdose cases (Bohler et al. 2019; Krauskopf et al. 2015). APAP overdose is a leading cause of acute liver failure (ALF) in high-income countries, but its global burden is poorly defined due to underreporting and limited surveillance in low-resource settings. Recent estimates indicate that APAP is implicated in approximately 6% of poisoning cases worldwide, contributes to 56% of ALF presentations, and accounts for 7% of overall DILI cases (Chidiac et al. 2023). Despite its clinical importance, APAP hepatotoxicity research has relied extensively on mouse models that closely recapitulate human pathophysiology (Jaeschke et al. 2021). However, key APAP effects on human liver systems, particularly at single-cell resolution, remain insufficiently understood. Deeper insight into cell–cell communication among the diverse liver cell types will support the development of more advanced and human-relevant test systems. Concerns over the translational relevance and ethics of animal-based testing have driven a global shift towards next-generation risk assessment (NGRA) using New Approach Methodologies (NAMs).

Among NAMs, conventional in vitro systems, particularly two-dimensional (2D) monolayer cultures, have played a pivotal role in probing APAP-induced hepatotoxicity. Hepatic cell lines such as the human hepatocarcinoma cell line (HepG2) are widely utilized for initial mechanistic and toxicity screenings due to their ease of culture, reproducibility, and compatibility with high-throughput screening (Gerets et al. 2012; Godoy et al. 2013; Sison-Young et al. 2017). While widely used, HepG2 cells exhibit notable limitations, particularly their low expression of key cytochrome P450 enzymes (CYPs) such as CYP2E1, which is essential for APAP bioactivation (Gerets et al. 2012; Ramaiahgari et al. 2014). To mitigate these shortcomings, alternative in vitro models, including HepaRG cells, and induced pluripotent stem cell-derived hepatocyte-like cells (iPSC-HLCs) have been adopted. HepaRG cells offer enhanced metabolic functionality relative to HepG2 cells, including the inducible expression of key CYPs such as CYP3A4 and CYP2E1, thereby providing greater predictive utility for DILI assessments (DiProspero et al. 2022; Duivenvoorde et al. 2021; Lorincz et al. 2021). Even with improved functionality, the differentiation process for HepaRG cells is time-consuming, and their expression levels of key drug metabolizing enzymes may still fall short of in vivo conditions (Okuyama et al. 2022). Similarly, iPSC-HLCs offer a renewable platform with the potential for modeling genetic diversity and disease, but their application is limited by cellular immaturity, low cytochrome P450 activity, and inconsistent expression of hepatic markers (Ghosh et al. 2021; Jin et al. 2021).

To replicate native hepatic function with higher fidelity, primary human hepatocytes (PHHs) are widely used as the benchmark for in vitro liver models and the gold standard for assessing DILI. PHHs exhibit robust expression of drug metabolizing enzymes, reflect native hepatic architecture, and reliably recapitulate in vivo drug metabolism and toxicity responses (Gomez-Lechon et al. 2003; Weaver et al. 2020). Unlike HepG2 cells and other surrogate models, PHHs robustly express physiological levels of CYPs critical for the bioactivation of APAP to its toxic metabolite, N-acetyl-p-benzoquinone imine (NAPQI), enabling more accurate hepatotoxicity assessment (Negoro et al. 2022). However, PHH cultured in conventional 2D conformation rapidly dedifferentiate, leading to loss of hepatic function and limiting their utility for long-term DILI risk evaluation (Bell et al. 2017; Gupta et al. 2021; Heslop et al. 2017; Lauschke et al. 2016). These limitations prompted the development of three-dimensional (3D) liver models such as spheroids, organoids, and scaffold-based systems.

Compared to 2D cultures, 3D platforms better preserve cell polarity, intercellular interactions, and sustained metabolic activity, thereby improving the predictive performance of hepatotoxicity assays (Bell et al. 2016; Ramaiahgari et al. 2014; Underhill & Khetani 2018). These 3D liver models facilitate a more accurate simulation of APAP metabolism, oxidative stress responses, and zonal hepatotoxicity (Ahn et al. 2019; McGill et al. 2012), thereby enhancing the mechanistic understanding of APAP-induced liver injury. To further improve physiological relevance, 3D liver models have been co-cultured with non-parenchymal cells such as stellate cells, Kupffer cells (KCs), and endothelial cells, providing a more holistic view of APAP-induced hepatic injury (Bale et al. 2016; Godoy et al. 2013; Messner et al. 2018; Ware et al. 2018).

In parallel with advances in liver model complexity, transcriptomic technologies have evolved to enable deeper mechanistic insight into drug-induced hepatotoxicity. Early transcriptomic efforts relied on DNA microarrays, which facilitated large-scale gene expression profiling and supported the identification of compound-specific and temporal response signatures (Chin & Kong 2002; Neumann & Galvez 2002; Zhang et al. 2014). The advent of bulk RNA sequencing (RNA-seq) enabled more sensitive and comprehensive profiling of transcriptomes, uncovering global hepatic perturbations associated with xenobiotic metabolism. For instance, RNA-seq has been utilized to quantify the mRNA abundance of major xenobiotic-processing genes in human liver and intestine, revealing tissue-specific expression patterns and aiding in the understanding of inter-individual variability in drug metabolism (Fu et al. 2016). In a related application, Gupta et al. (2021) applied RNA-seq to evaluate the transcriptomic similarity of various in vitro human liver models to native liver tissue, with particular emphasis on xenobiotic metabolism pathways. Their analysis revealed that certain 3D liver microtissues and PHH closely mirrored in vivo gene expression profiles, underscoring the value of RNA-seq in benchmarking liver models for toxicological applications. These studies have successfully identified differentially expressed genes (DEGs) and regulatory networks that not only illuminate the progression of hepatotoxicity but also provide valuable biomarkers for early DILI detection. Yet despite these successes, bulk RNA-seq captures only averaged signals across heterogeneous tissues, limiting its ability to resolve the distinct transcriptional responses of individual cell types.

In addressing the limitation of bulk RNA-seq, this study employed single-cell RNA sequencing (scRNA-seq) to characterize the transcriptional landscape of APAP-exposed liver microtissues. These microtissues comprised PHHs, KCs, and hepatic sinusoidal endothelial cells (HSECs) cultured as 2D monolayers or 3D spheroids. Guided by NGRA principles we aimed to determine which cell-culture architecture best captures liver-specific responses to APAP exposure at single-cell resolution. Notably, our preliminary analyses revealed a striking enrichment of hypoxia-associated gene expression in 3D microtissues. Given that APAP bioactivation by CYP enzymes, primarily CYP2E1, CYP1A2, and CYP3A4, involves substantial oxygen consumption (Gehre et al. 2020; Ramachandran & Jaeschke 2020), we posited two hypotheses:

i. 3D architecture intrinsically induces a hypoxic baseline due to limited oxygen diffusion, or
ii. APAP exposure exacerbates hypoxia-like stress responses within the microtissue core

By systematically comparing single-cell transcriptional profiles across control (CTRL), low-dose (LD), and high-dose (HD; evaluated exclusively in 3D) conditions, this study seeks to elucidate the interplay between tissue architecture, drug exposure, and cellular stress responses, thereby informing the selection of optimal in vitro models for human-relevant assessment of DILI.

## Methods

### Cell culture

Cryopreserved pooled plateable PHHs, HSECs and male human KCs were obtained from Tebubio. All cryopreserved liver cells were thawed according to the suppliers’ instructions and resuspended in maintenance media (William’s E media with 2 mM L-glutamine, 100 U/ml penicillin, 100 μg/ml streptomycin, 10 μg/ml insulin, 5.5 μg/ ml transferrin, 6.7 ng/ml sodium selenite, 100 nM dexamethasone) supplemented with 20% foetal bovine serum (FBS). For 3D experiments, spheroids were established by seeding 1000 PHHs, 185 KCs and 250 HSECs per well in ultra-low attachment plates and centrifuged for 2 min at 125 × g. After 3 days media was changed to FBS-free maintenance media and cultures were maintained until day 5 post-seeding, when APAP exposure was initiated. For 2D experiments, 24-well plates (Gibco) were precoated with 5 µg/cm2 rat tail collagen type 1 in 0.02 M acetic acid for 1 h at room temperature and washed 3 times with PBS. PHHs, KCs and HSECs in the same ratio as for 3D were seeded at 350,000 cells per well and allowed to attach for 4 h at 37 °C in a humidified incubator. Subsequently, debris was removed by shaking and washing the cells twice with maintenance media and cells were covered with 150 μL/well of 2 µg/mL collagen-mixture in DMEM and incubated at 37 °C for approximately 30 min until the collagen was fixed. Finally, media was replaced for maintenance media and cultures were maintained until day 5 post-seeding, when APAP exposure was initiated.

The 3D liver spheroids were exposed for 24 h to APAP at concentrations of 350 µM (LD) and 2687 µM (HD), while 2D sandwich cultures were exposed only to LD APAP. Both 2D and 3D models were exposed to milli-q as vehicle control.

### Library preparation and sequencing

After 24 h of exposure for 3D cultures, 192 spheroids per condition were collected in 900 microL PBS, washed and then resuspended in Liberase TL Research Grade™ 1.3 U/mL (Roche; 5,401,020,001). For 2D, cells were treated with the same liberase digestion mix using 500 µL per well. The mixture was incubated at 37ºC for 30 min shaking @100 RPM, with additional manual mixing by vortex every 10 min. Dissociation into single cells was checked using a microscope. Dissociated spheroids were kept on ice. Cells were manually counted using Trypan blue and resuspended in PBS + 0.04% BSA at a concentration of 1,000 cells/µL to recover ∼ 10,000 cells per sample.

Single-cell RNA-Seq libraries were generated from cells using the Chromium™ Next GEM Single Cell 3′ Kit v3.1 (10X Genomics, PN-1000269) and the Dual Index Kit TT Set A, 96 reactions (10X Genomics, PN-1000215), following the manufacturer’s protocol. Library concentration was measured with a Qubit 2.0 Fluorometer (ThermoFisher), and quality was assessed using an Agilent BioAnalyzer 2100. Sequencing was carried out on an Illumina NovaSeq 6000 system using a 100-cycle S1 flow cell (v1.5) in paired-end mode.

### Data pre-processing in cell ranger

Raw sequencing data were processed using the 10 × Genomics Cell Ranger 7.0.0 pipeline to generate cell barcodes, perform read alignment, and quantify gene expression. Briefly, raw binary base call (BCL) files were demultiplexed into fastq files using cellranger mkfastq. The generated fastq files were aligned to the human reference genome (http://cf.10xgenomics.com/supp/cell-exp/refdata-cellranger-GRCh38-1.2.0.tar.gz) using cellranger count, with the –force-cells 5000 option applied to retain a minimum of 5,000 cells for analysis. The resulting gene expression matrices for each of the samples were imported into Seurat version 5.1.0 (Hao et al. 2023) for quality control (QC) and further downstream analysis.

### Data processing in Seurat

Cells with fewer than 800 unique molecular identifier (UMI) counts or fewer than 500 detected genes were filtered out. The expression of each sample was log-normalized before merging the samples into a single Seurat object for each cell conformation (2D and 3D). Cells with similar expression patterns were clustered using a resolution of 1.2. The resulting clusters were annotated using the ScType algorithm (Ianevski et al. 2022) with a selection of cell-specific gene markers from either Panglaodb (Franzen et al. 2019) or CellMarker2.0 (Hu et al. 2023) for expected cell types in our cultures. DEGs were identified using a false discovery rate (FDR) threshold of < 0.05 by comparing gene expression profiles of the same cell types between 2 and 3D cultures under baseline conditions. The DEGs identified for each cell type (Supplementary File 2) were pooled into a single list and used as input for pathway enrichment analysis using the Reactome database (Milacic et al. 2024). To provide an overview of affected biological processes, the Reactome hierarchical pathway structure was used to organize enriched pathways. Only the two highest levels of the pathway hierarchy were visualized, with top-level pathways indicated by black boxes. The full code used for this analysis is available at https://github.com/TGX-UM/APAP_scRNA-seq/tree/main/Script.

### Hypoxia gene marker list

We investigated hypoxic responses at the single-cell level using a curated list of 16 hypoxia marker genes derived from gene expression studies on liver cells exposed to low oxygen levels (Koritzinsky et al. 2013). We applied this hypoxia marker list to annotate individual cells, enabling us to assess the expression of these markers within each cell type and visualize the clustering patterns associated with hypoxic responses across different cell culture conformations.

### CTD APAP gene list

A comprehensive list of APAP-responsive genes was obtained from the Comparative Toxicogenomics Database (CTD) (Davis et al. 2023) and subsequently refined using the following filtering criteria to include only verified genes:

i. Only entries for acetaminophen (Chemical name: Acetaminophen, Chemical ID: D000082, CAS RN: 103–90-2) were retained
ii. Genes with documented evidence of involvement in humans, as well as in at least one additional species were included in the analysis
iii. Interaction actions such as affects^expression, decreases^expression, increases^expression, and their combinations (e.g., increases^expression|increases^reaction) were selected
iv. Only interactions related to mRNA were considered
v. Entries with fewer than 10 references were removed

This filtering yielded a curated list of 20 verified APAP-responsive genes, which are detailed in Supplementary Table 1.

###  Data availability

All raw scRNA-seq data generated in this study have been deposited in the BioStudies database under accession number E-MTAB-15516.

## Results

scRNA-seq profiled human liver microtissues comprising PHHs, HSECs, and KCs, cultured as 2D monolayers or 3D spheroids. Baseline transcriptional states were defined to control for architectural effects and establish reference profiles for interpreting subsequent perturbations. APAP exposure was then assessed across CTRL, LD, and HD conditions, the latter evaluated only in 3D, to quantify architecture- and dose-dependent single-cell responses. The results below detail how culture architecture and APAP dosing shape hepatic transcriptional responses at single-cell resolution.

### scRNA-seq reveals baseline transcriptional differences between 2 and 3D liver cell cultures

**Fig. 1:**
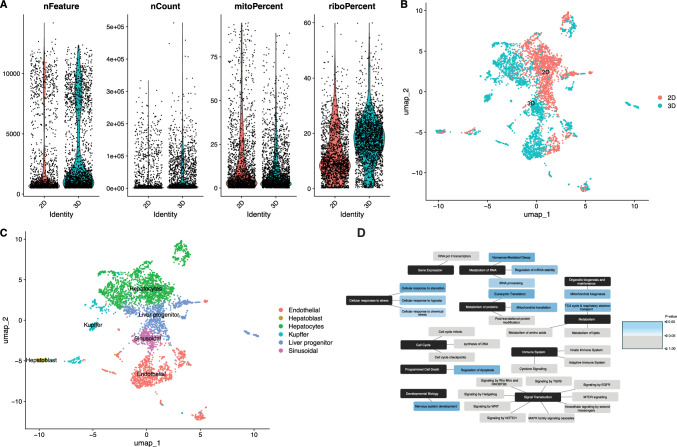
Baseline transcriptional and pathway-level differences between 2 and 3D liver cell cultures identified by scRNA-seq

To characterize baseline transcriptional diversity and RNA content across the two cell culture conformations, we analyzed scRNA-seq data from CTRL samples. Following the criteria for QC described in methods, 1,849 cells from 2D culture and 2,157 from 3D culture were retained for downstream analysis. Violin plots of unique genes per cell (nFeature) showed higher median gene counts and greater variability in 3D cultures, suggesting increased transcriptional diversity compared to 2D cultures (Fig. 1a). Total RNA content per cell (nCount) was also higher and more variable in 3D cultures compared to 2D, supporting enhanced transcriptional output and cellular heterogeneity in 3D microtissues (Fig. 1a). Both culture formats showed low mitochondrial read percentages (mitoPercent), with most cells below the 10% threshold, indicating good cell viability (Fig. 1a). Ribosomal content (riboPercent) was elevated and more variable in 3D culture (Fig. 1a), suggesting increased translational activity or shifts in cellular state compared to 2D culture. Supplementary Fig. 1a provides a summary of cells retained post-QC across all dose conditions.

Uniform Manifold Approximation and Projection (UMAP) revealed distinct clustering patterns between 2 and 3D cultures (Fig. 1b), suggesting transcriptional divergence driven by culture architecture. We also observed areas of overlap between cell clusters, suggesting shared gene expression profiles among corresponding cell types in both 2D and 3D cultures (Fig. 1b-c). Differential expression analysis between matched cell types revealed transcriptional differences between the 2D and 3D cultures. The number of DEGs identified between 2 and 3D cultures for each cell type is summarized in Table 1. Notably, only a single DEG, *CXCR4*, was detected among hepatoblasts, with higher expression in the 3D compared to the 2D conformation. Endothelial cells exhibited the largest transcriptional shift, with 3,745 DEGs, representing the highest number among all cell types analyzed. Supplementary File 2 lists all DEG’s for each cell type.

**Table 1:** Number of DEGs identified between unexposed 2D and 3D cell cultures (FDR < 0.05) across corresponding cell types

Cell type	DEGs between 2 and 3D cells in baseline CTRL condition
Endothelial cells	3745
Sinusoidal cells	14
Hepatocytes	770
Liver progenitor cells	898
Hepatoblast	1
Kupffer cells	1659

### Reactome analysis identifies hypoxia-related stress responses in 3D liver cultures

Mechanistic insight into the transcriptional differences between 2 and 3D liver cultures under baseline conditions was obtained by pooling DEGs from matched cell types and analyzing them through Reactome pathway enrichment (Milacic et al. 2024). As expected, several pathways commonly associated with in vitro culture systems were enriched. However, a striking finding was the significant enrichment of stress-related pathways, most notably, the cellular response to hypoxia pathway (Fig. 1d), which reached statistical significance (p < 0.05).

**A** Violin plots comparing four key quality control metrics across 2D and 3D cultures. nFeature represents the number of unique genes detected per cell, nCount indicates total UMI counts per cell, mitoPercent reflects the percentage of reads mapped to mitochondrial genes, and riboPercent denotes the percentage of reads mapped to ribosomal protein-coding genes. Each dot represents a single cell.** B** UMAP visualization of liver cells from unexposed 2D and 3D cultures, showing distinct transcriptional clustering with regions of overlap indicating shared gene expression profiles among matched cell types. **C** UMAP displaying cell type annotations derived from cluster identities across both 2D and 3D samples. **D** Reactome pathway enrichment results based on pooled DEGs between 2 and 3D cultures, highlighting significant enrichment of hypoxia-related pathways in 3D liver microtissues.

### Cell type specific annotation of hypoxic responses in CTRL cultures using marker gene expression

**Fig. 2:**
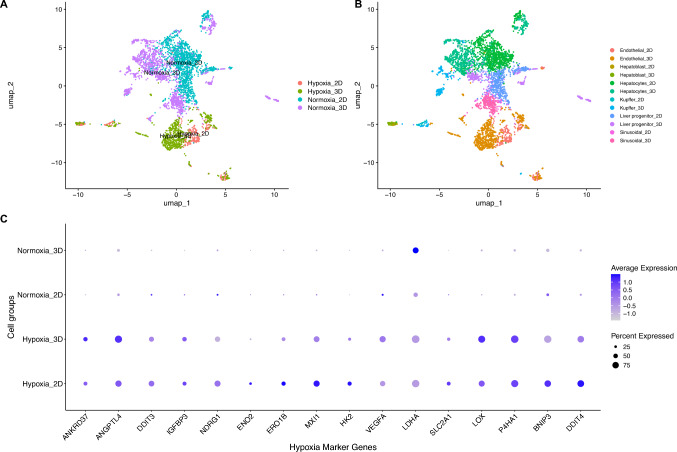
Hypoxia marker gene expression in control 2D and 3D liver cultures.** A** UMAP showing clustering of cells annotated based on hypoxia marker expression. Cells expressing hypoxia-associated genes are labeled as Hypoxia_2D or Hypoxia_3D, while those lacking these markers are labeled as Normoxia_2D and Normoxia_3D. **B** UMAP showing the distribution of individual cell types in 2D and 3D cultures after annotation with hypoxia markers. Each point represents a single cell, colored according to its annotated cell type as indicated in the legend.** C** Dot plot summarizing hypoxia marker expression across 2D and 3D CTRL liver cultures. Dot size indicates the percentage of cells expressing each gene; color indicates average expression

Building on the observation of hypoxia-related pathway enrichment, we investigated cell-type–specific expression of hypoxia marker genes across 2D and 3D liver cultures under CTRL conditions. A curated panel of 16 hypoxia-responsive genes, based on gene expression profiling of hepatocytes exposed to low oxygen levels was used to annotate single-cell clusters and evaluate the distribution of hypoxia-related transcriptional activity. UMAP visualization (Fig. 2a and b) displays the clustering patterns of cells annotated for hypoxia-associated gene expression in both 2D and 3D liver cultures. Cells with elevated expression of known hypoxia marker genes formed distinct clusters in both conditions, with a higher density observed in the 3D cultures. These hypoxia-enriched cells were predominantly observed in endothelial and hepatoblast populations, indicating that these cell types are more transcriptionally responsive to low-oxygen conditions in the 3D microenvironment.

Expression of the liver-specific hypoxia markers was then examined across annotated cell populations to gain further insight into the transcriptional features of hypoxic cells. Cells from the 3D culture conformation exhibiting a hypoxic signature demonstrated markedly elevated expression levels of well-established hypoxia-responsive genes, including *LDHA*, *VEGFA*, *ANGPTL4*, *LOX*, and *P4HA1* (Fig. 2c). While both 2D and 3D cells expressed *ANGPTL4*, *LOX*, and *P4HA1*, expression levels were consistently higher in 3D cultures. Interestingly, cells from the 2D culture also displayed elevated expression of select hypoxia markers, including *MXI1*, *DDIT4*, *BNIP3*, *ENO2*, *HK2*, and *ERO1B,* compared to their expression in 3D cultures. However, the proportion of 2D cells expressing these genes at high levels remained below 50%, indicating a less uniform or less intense hypoxic response. Cells classified as normoxic displayed minimal expression of hypoxia marker genes, consistent with their designation. An exception was *LDHA*, which remained conspicuously expressed in both normoxic groups, with higher expression observed in 3D cultures. Additionally, a small subset (< 25%) of normoxic 2D cells exhibited trace expression of *VEGFA*, *NDRG1*, *DDIT3*, and *BNIP3*, indicating faint hypoxia-associated transcription in a limited population of cells. A summary of the expression patterns for these hypoxia-associated genes across cell populations is presented in the dot plot shown in Fig. 2c. Supplementary Fig. 3 presents the expression profiles of additional candidate hypoxia-marker genes used to annotate cells in both 2D and 3D culture models.

### Transcriptional responses to APAP exposure in 2D and 3D liver cell Cultures

**Fig. 3:**
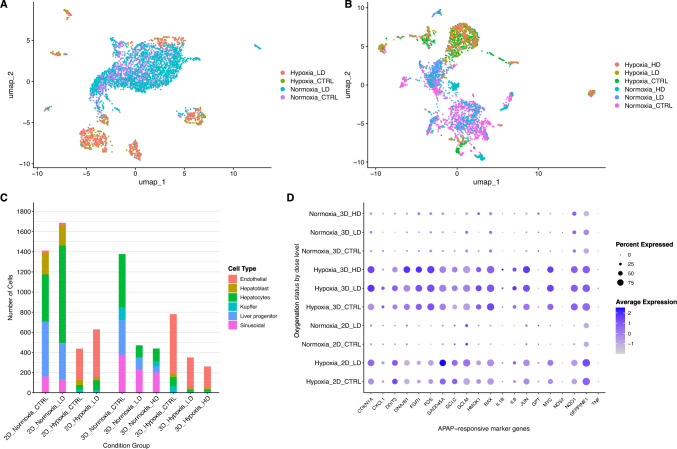
**A–B** UMAP visualizations showing cell annotations based on hypoxia marker gene expression across APAP dose conditions in 2D and 3D liver cell culture models, respectively. **C** Stacked bar chart showing the distribution of annotated liver cell types across CTRL, LD, and HD samples, separated by oxygenation status and culture format (2D vs. 3D). **D** Dot plot showing the expression levels of 20 known APAP-responsive genes across 2D and 3D liver cell cultures under CTRL, LD, and HD APAP conditions

Following baseline characterization, we evaluated APAP exposure effects at single-cell resolution across 2D and 3D liver microtissues. Analyses tested for transcriptional signatures consistent with APAP metabolic bioactivation and quantified changes in hypoxia-associated cell populations across all dose conditions (CTRL, LD, and HD; 3D only) in each culture conformation (Supplementary Fig. 1a). In every condition, normoxic cells outnumbered hypoxic cells in both 2D and 3D (Supplementary Fig. 1b). The proportion of hypoxic cells was highest in 3D CTRL culture (Supplementary Fig. 1b). On average, 38.7% of cells in 3D culture expressed hypoxia marker genes, compared to 25.4% of cells in 2D culture (Supplementary Figs. 1b–f), a significant difference (p = 0.019, two-sided t-test). Beyond differences in overall proportions, distinct cell-type distributions were observed between hypoxic and normoxic populations (Fig. 3a–c). Normoxic cell populations included most PHHs, as well as all HSECs and liver progenitor cells. In contrast, hypoxic populations were largely composed of endothelial cells.

In 2D cultures, LD APAP exposure increased the proportion of hypoxia-positive cells to 27.1%, compared to 23.6% under CTRL conditions (Fig. 3c), indicating a shift in hypoxia-associated representation. Endothelial cells were minimally represented within the normoxic population of 2D cells, irrespective of treatment, and were completely absent from normoxic populations in 3D cultures (Fig. 3c). Similarly, hepatoblasts were undetected among normoxic cells in 3D cultures (Fig. 3c). Neither HSECs nor liver progenitor cells expressed hypoxia markers under any exposure condition (Fig. 3c). Notably, in 3D cultures, hypoxia-positive Kupffer cells, hepatocytes, hepatoblasts, and endothelial cells were most abundant under CTRL conditions (> 700 cells) but declined sharply after APAP exposure, with fewer than 400 hypoxia-positive cells in both LD and HD conditions (Fig. 3c).

To assess APAP uptake in 2D and 3D liver models, we examined the expression profiles of 20 APAP-responsive genes selected based on criteria outlined in the methods section. As expected, cells from both 2D and 3D CTRL conditions generally exhibited low expression of these genes, consistent with no APAP exposure (Fig. 3d). Across all dose conditions, normoxic cells in both 2D and 3D cultures consistently exhibited low expression of APAP-responsive marker genes (Fig. 3d). In contrast, hypoxic 3D populations displayed elevated average expression of these genes, with moderate expression already present under CTRL conditions and further increased upon APAP exposure. A similar, though less pronounced, pattern was observed in hypoxic 2D populations, where APAP-responsive genes showed moderate expression in CTRL and were modestly induced under LD exposure. Notably, hypoxic 3D cells demonstrated a dose-dependent response, as *DNAJB1*, *EGR1*, *FOS*, and *MYC* exhibited higher average expression under HD compared to LD conditions (Fig. 3d).

In both 2D and 3D cultures, Kupffer cells annotated as hypoxic exhibited high expression of *HMOX1* and *GCLC* in over 50% of cells (Supplementary Fig. 2). In hypoxic endothelial cells from 3D cultures, elevated levels of *IL6*, *NQO1*, *MYC*, *EGR1*, *FOS*, *SERPINE1*, and *BAX* were observed. Conversely, in normoxic endothelial cells from 2D cultures, *SERPINE1*, *EGR1*, *CXCL1*, and *GCLC* were highly expressed (Supplementary Fig. 2). An exhaustive breakdown of gene expression by cell type, dose, and oxygenation status is provided in Supplementary Fig. 2.

### The intersect between hypoxia and the expression of key APAP metabolism genes

**Fig. 4:**
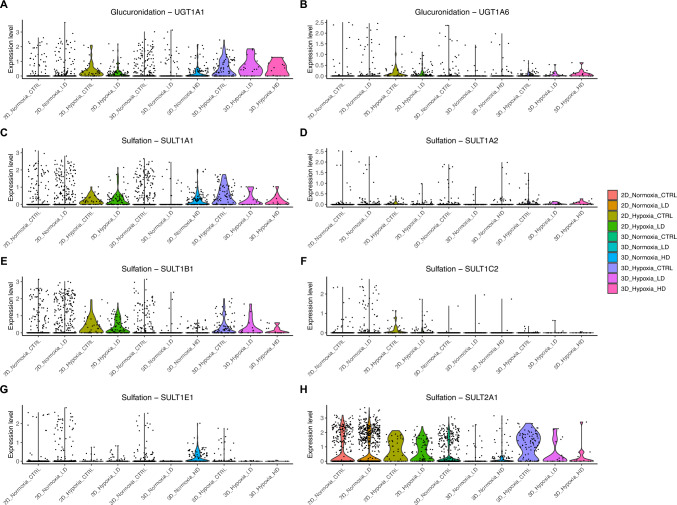
Expression of APAP metabolism-associated genes in hepatocytes across 2D and 3D cultures. **A–B** Violin plots showing the expression levels of *UGT1A1* and *UGT1A6*, enzymes involved in the glucuronidation of APAP. **C–H** Violin plots showing expression of sulfotransferases (*SULT1A1*, *SULT1A2*, *SULT1B1*, *SULT1C2*, *SULT1E1*, *SULT2A1*) responsible for APAP sulfation. The color legend (left) indicates the order and identity of sample groups on the x-axis

We investigated whether hypoxia alters the expression of genes central to APAP metabolism in hepatocytes, and whether APAP metabolism itself contributes to hypoxia within hepatocytes. These APAP metabolism genes were selected based on McGill and Jaeschke (2013). During the initial phase of APAP clearance, the drug undergoes glucuronidation and sulfation before excretion. Hepatocytes expressed *UGT1A1* and *UGT1A6*, key enzymes mediating glucuronidation in humans (Fig. 4a–b). Both genes displayed elevated expression under hypoxic conditions relative to normoxic hepatocytes, with the strongest effect observed in 3D culture. In 3D hypoxic hepatocytes, cell numbers decreased under HD compared to LD and CTRL conditions. UGT1A1 expression also declined with increasing APAP dose (Fig. 4a). A similar dose-dependent decline was observed in 2D hypoxic hepatocytes, where UGT1A1 expression was higher in CTRL relative to LD APAP exposure (Fig. 4a). Consistent with this trend, multiple sulfotransferases (*SULT1A1*, *SULT1A2*, *SULT1B1*, and *SULT2A1*) showed higher expression in hypoxic hepatocytes compared to normoxic cells (Fig. 4c–h).

**Fig. 5:**
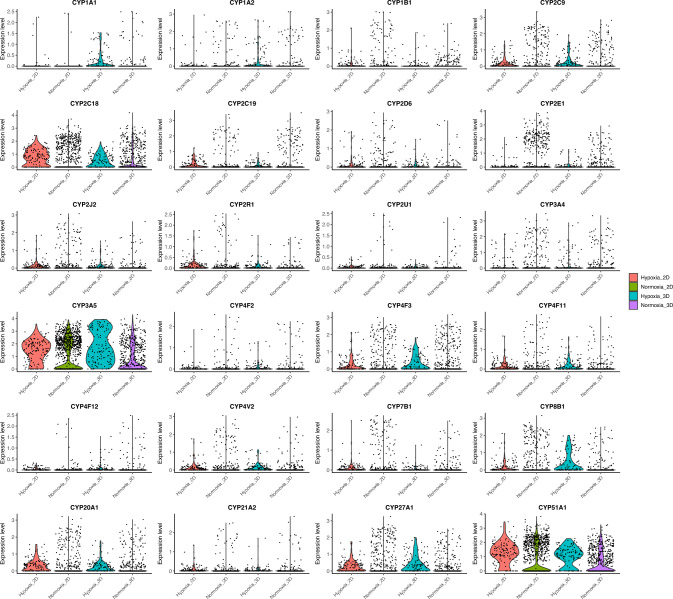
Violin plots showing CYP gene expression in hepatocytes across hypoxic and normoxic cells in both 2D and 3D conformation

APAP that is neither glucuronidated nor sulfated is metabolized by cytochrome P450 enzymes, generating the reactive intermediate NAPQI. We therefore examined CYP expression patterns across hypoxic and normoxic hepatocytes in both 2D and 3D cultures (Fig. 5). CYP1A1 expression was moderate in both hypoxic and normoxic 3D hepatocytes and reduced in hypoxic and normoxic 2D cultures. CYP1A2 was highly expressed in normoxic 2D, hypoxic 3D and normoxic 3D hepatocytes, whereas hypoxic 2D cells showed the lowest levels. CYP1B1 expression was highest in normoxic 2D hepatocytes and moderate in normoxic 3D cells. Within the CYP2 family, CYP2C9 was more abundant in normoxic 2D and normoxic 3D hepatocytes than in either hypoxic condition. CYP2C18 was generally highly expressed across all groups but was decreased in hypoxic hepatocytes in both 2D and 3D. CYP2C19 was elevated in normoxic 3D and normoxic 2D cells, and CYP2D6 expression was abundant in normoxic 2D hepatocytes. CYP2E1 levels were highest in normoxic 2D and normoxic 3D cultures, with both hypoxic groups exhibiting the lowest expression. CYP2J2 expression was greater in normoxic 2D cells and lowest in hypoxic 2D and 3D hepatocytes. CYP2R1 showed a subset of hepatocytes with high expression under normoxic 2D conditions. By contrast, CYP2U1, CYP21A2 and CYP4F2 were overall lowly expressed, with only occasional cells showing higher expression in normoxic 2D and normoxic 3D cultures. Among CYP3 and related genes, CYP3A4 was highly expressed in normoxic 2D and normoxic 3D hepatocytes and reduced in both hypoxic conditions. CYP3A5, CYP51A1, CYP27A1, CYP4F3 and CYP8B1 exhibited consistently high expression across all four conditions, with CYP3A5 showing the highest overall levels. CYP7B1 expression was higher in normoxic hepatocytes than in hypoxic counterparts.

## Discussion

This study presents a single-cell transcriptomic analysis of APAP-induced hepatotoxicity in human liver microtissues, revealing how tissue culture architecture shapes cellular responses to drug exposure. By comparing PHH co-cultures in 2D monolayers and 3D spheroids, we demonstrate that 3D cultures exhibit greater transcriptional complexity, higher cellular RNA content, and increased variability in ribosomal gene expression. These features reflect heightened metabolic and biosynthetic activity and align with prior reports showing that 3D hepatic spheroids better preserve hepatocyte maturity, functionality, and longevity relative to 2D systems (Brazovskaja et al. 2019; Morell et al. 2024). In parallel with these transcriptional differences, Reactome pathway analysis identified significant enrichment of hypoxia-associated signaling in baseline 3D culture. This likely reflects limited oxygen diffusion into the spheroid core, which can lead to non-physiological hypoxia in deeper layers of the spheroid. A phenomenon that has been reported to occur in 3D spheroid cultures even at 21% oxygen levels (Gomes et al. 2016; Murphy et al. 2023; Tse et al. 2021). Importantly, although the pericentral region (zone 3) of the liver has a lower oxygen tension than the periportal region, it is not hypoxic under physiological conditions, as each hepatocyte borders one or more sinusoids that ensure adequate oxygen supply. Thus, the hypoxic signatures observed in 3D spheroids arise from diffusion limitation, where oxygen must pass through multiple layers of oxygen-consuming cells, rather than representing true in vivo centrilobular oxygen levels. Nevertheless, the presence of a relative oxygen gradient in the 3D cultures provides a relative spatial cue that parallels aspects of hepatic zonation (Kietzmann 2017; Paris & Henderson 2022; Wesseler et al. 2023). This gradient not only regulates hepatocyte function but also affects the localization and behavior of non-parenchymal cells. In our dataset, endothelial cells displayed the strongest hypoxia-associated transcriptional profile, with widespread expression of canonical hypoxia markers (Fig. 3c). Since endothelial cell responsiveness to oxygen gradients is well documented in the context of angiogenesis and vascular remodeling (Krock et al. 2011), we considered the possibility that this signal reflected gene set overlap between endothelial markers and hypoxia-associated genes. However, direct comparison revealed no such overlap, supporting the interpretation that the observed expression signature reflects a bona fide hypoxic response. Taken together, these results suggest that 3D liver spheroids establish relative oxygen gradients capable of inducing zonation-like transcriptional patterns. Future methods offering more precise regulation of oxygen diffusion could permit the establishment of gradients that more closely resemble the physiological periportal-to-pericentral oxygen levels.

Our findings point to a bidirectional relationship between hypoxia and APAP metabolism, whereby oxygen availability shapes cellular metabolic responses, and APAP biotransformation, in turn, influences the hypoxic landscape within liver microtissues. Quantitative shifts in cell counts across different oxygenation states and exposure conditions underscore this dynamic interplay (Fig. 3c–d). In 2D cultures, normoxic hepatocytes exposed to LD APAP increased in number relative to CTRL, suggesting that APAP metabolism may initially promote hepatocyte survival or proliferation in well-oxygenated conditions. This effect may reflect a transient activation of cytoprotective Nrf2 signaling by NAPQI, which is known to induce Nrf2 as an adaptive defense mechanism against oxidative stress and mitochondrial dysfunction (Dong et al. 2025; Jiang et al. 2016). In contrast, cells within hypoxic 2D environment remained fewer in number, with only modest increases following APAP exposure, highlighting a possible limitation in metabolic or proliferative capacity under oxygen-restricted conditions.

In 3D cultures, the impact of APAP on hypoxia-annotated cells was more pronounced, as their numbers declined sharply with increasing APAP dose (Fig. 3c). Concurrently, APAP-activating CYPs such as CYP1A2, CYP2C9, CYP2E1 and CYP3A4 were preferentially expressed in normoxic hepatocytes, whereas CYP3A5 remained highly expressed across both normoxic and hypoxic states (Fig. 5). These patterns suggest that, in our spheroid model, APAP bioactivation occurs predominantly in oxygenated hepatocytes, where CYP-mediated catalysis consumes molecular oxygen. Such activity may further steepen diffusion-limited oxygen gradients between normoxic and hypoxic regions (Gehre et al. 2020; Hinson et al. 2004; Jaeschke et al. 2012). The resulting exacerbation of hypoxia may impair phase II detoxification capacity, promoting cellular stress and loss. Supporting this, we observed that *UGT1A1* and *SULT1A1* (Fig. 4a and c), key enzymes for glucuronidation and sulfation, respectively, were most highly expressed in hypoxic hepatocytes under control conditions but declined with APAP exposure. This pattern may reflect either exhaustion of detoxification capacity or hypoxia-induced transcriptional reprogramming (Lin et al. 2012). Collectively, these data suggest that APAP metabolism can exacerbate hypoxic stress in liver microtissues, particularly in 3D cultures where oxygen diffusion is intrinsically limited, and that hypoxia, in turn, modulates the expression of metabolic enzymes, revealing a reciprocal feedback loop between oxygen tension and drug metabolism.

The complete absence of endothelial cells, and similarly hepatoblasts from normoxic populations in 3D cultures can be attributed to a combination of biological localization and technical annotation factors. First, 3D spheroids inherently develop oxygen gradients due to diffusion limitations, with the core regions becoming hypoxic as spheroid size increases (Riffle & Hegde 2017). Endothelial cells and hepatoblasts may preferentially localize to these low-oxygen niches, consistent with known endothelial adaptations to hypoxia, such as the activation of angiogenic and metabolic transcriptional programs (Vorwald et al. 2020). Second, the process of cell-type annotation using scType, which assigns identities based on a weighted scoring scheme for positive and negative marker genes, may further contribute to their apparent absence. Under hypoxic stress, these cell types may downregulate classical identity markers in favor of hypoxia-inducible or stress-responsive genes, which are not typically included in canonical marker sets. As a result, such cells may fall below the classification threshold set by scType and remain unannotated or misclassified. This limitation is particularly relevant in single-cell transcriptomic datasets, where transcriptional plasticity under environmental perturbations, combined with gene dropout and low expression of markers, can obscure cell identity and reduce annotation accuracy (Ianevski et al. 2022). We acknowledge that all interpretations in this study are based on transcriptional changes, which may not directly correspond to protein abundance or functional effects. Indeed, it has long been recognized that the correlation between transcriptomic and proteomic profiles is generally poor (Ghazalpour et al. 2011; Schwanhausser et al. 2011). Accordingly, the gene expression patterns we report should be viewed as indicative rather than definitive evidence of mechanistic involvement in APAP-induced injury. Additional proteomic or functional validation will be required to determine the pathophysiological relevance of these transcriptional responses in vivo.

Although hepatoblasts and liver progenitor cells were not intentionally introduced into the PHH co-cultures at the time of seeding, their emergence as distinct transcriptional clusters may reflect cellular plasticity and de-differentiation processes known to occur in in vitro liver models. Under culture stress, oxidative imbalance, or exposure to xenobiotics such as APAP, mature hepatocytes can undergo phenotypic reprogramming and acquire progenitor-like features (Tarlow et al. 2014; Yanger et al. 2013). Furthermore, lineage ambiguity in scRNA-seq is a recognized phenomenon. In stressed or transitioning states, hepatocytes may downregulate classical identity markers, and transiently express progenitor-associated transcripts (Gribben et al. 2024), causing the scType clustering algorithm to assign them to hepatoblast or liver progenitor cells. Importantly, these cells may not represent distinct progenitor populations per se, but rather adaptive states of hepatocytes or other epithelial cells in response to environmental perturbations.

## Conclusion

This study demonstrates that tissue architecture critically shapes the transcriptional landscape of human liver microtissues in response to APAP, revealing a dynamic interplay between oxygen availability and drug metabolism. Our findings highlight how 3D culture formats establish hypoxic gradients that in the future could be used to recapitulate hepatic zonation, thereby influencing both baseline cellular states and responses to xenobiotic challenge. By leveraging single-cell transcriptomics, we identified cell type–specific signatures of APAP metabolism and hypoxic stress, underscoring the mechanistic complexity underlying dose-dependent hepatotoxicity. While this work provides valuable insight into the reciprocal relationship between oxygen tension and metabolic activity, certain limitations remain. Notably, the high cost and sequencing requirements of scRNA-seq constrained the scope of our experimental design. Specifically, only LD APAP was evaluated in 2D cultures, precluding direct comparisons with HD conditions. Moreover, although our model includes multiple hepatic cell types, it lacks additional non-parenchymal populations such as cholangiocytes or hepatic stellate cells, which are known to play important roles in hepatic injury progression and regeneration. Incorporating these cell types, along with longitudinal and metabolomic profiling, could further refine the physiological relevance and mechanistic resolution of this platform.

These insights collectively reinforce tissue architecture as a critical consideration in the design and evaluation of NAMs for NGRA. Human 3D co-culture systems that maintain zonation-like oxygen gradients offer an enhanced platform for predictive toxicology, enabling more accurate, mechanistically anchored, and human-relevant DILI assessments.

## Supplementary Information

Below is the link to the electronic supplementary material.

Supplementary file1 (DOCX 3860 KB)

Supplementary file2 (XLSX 543 KB)
