# Supplementary material for: Single-cell transcriptomics of acetaminophen-induced responses in human 2D and 3D liver microtissues
Source: Arch Toxicol. 2026 Jan 14;100(4):1411–24. doi: 10.1007/s00204-025-04296-6 (PMC13043593; doi:10.1007/s00204-025-04296-6)
Supplement: Supplementary file 1 — Supplementary file1 (DOCX 3860 KB) [file 204_2025_4296_MOESM1_ESM.docx]

**Supplementary Tables and Figures**

**Supplementary Table 1:** Curated list of 20 verified acetaminophen (CAS No: 103-90-2, Chemical ID: D000082)-responsive genes obtained from the Comparative Toxicogenomics Database (CTD), following stringent filtering criteria.

| **Gene Symbol** | **Gene ID** | **Interaction** | **Interaction**  **Actions** | **Reference Count** | **Organism Count** |
| --- | --- | --- | --- | --- | --- |
| CDKN1A | 1026 | Acetaminophen results in increased expression of CDKN1A mRNA | increases^expression | 13 | 3 |
| CXCL1 | 2919 | Acetaminophen results in increased expression of CXCL1 mRNA | increases^expression | 18 | 2 |
| DDIT3 | 1649 | Acetaminophen results in increased expression of DDIT3 mRNA | increases^expression | 14 | 3 |
| DNAJB1 | 3337 | Acetaminophen results in increased expression of DNAJB1 mRNA | increases^expression | 10 | 3 |
| EGR1 | 1958 | Acetaminophen results in increased expression of EGR1 mRNA | increases^expression | 12 | 3 |
| FOS | 2353 | Acetaminophen results in increased expression of FOS mRNA | increases^expression | 12 | 3 |
| GADD45A | 1647 | Acetaminophen results in increased expression of GADD45A mRNA | increases^expression | 13 | 3 |
| GCLC | 2729 | Acetaminophen results in increased expression of GCLC mRNA | increases^expression | 27 | 3 |
| GCLM | 2730 | Acetaminophen results in increased expression of GCLM mRNA | increases^expression | 14 | 3 |
| HMOX1 | 3162 | Acetaminophen results in increased expression of HMOX1 mRNA | increases^expression | 37 | 3 |
| BAX | 959 | Acetaminophen results in increased expression of BAX mRNA | increases^expression | 10 | 3 |
| IL1B | 3553 | Acetaminophen results in increased expression of IL1B mRNA | increases^expression | 28 | 2 |
| IL6 | 3569 | Acetaminophen results in increased expression of IL6 mRNA | increases^expression | 23 | 3 |
| JUN | 3725 | Acetaminophen results in increased expression of JUN mRNA | increases^expression | 17 | 3 |
| GPT | 4552 | Acetaminophen results in increased expression of GPT mRNA | increases^expression | 11 | 3 |
| MYC | 4609 | Acetaminophen results in increased expression of MYC mRNA | increases^expression | 16 | 3 |
| NOS2 | 4843 | Acetaminophen results in increased expression of NOS2 mRNA | increases^expression | 13 | 2 |
| NQO1 | 1728 | Acetaminophen results in increased expression of NQO1 mRNA | increases^expression | 19 | 3 |
| SERPINE1 | 5054 | Acetaminophen results in increased expression of SERPINE1 mRNA | increases^expression | 12 | 3 |
| TNF | 7124 | Acetaminophen results in increased expression of TNF mRNA | increases^expression | 36 | 3 |

| **A** | **B**  **** |
| --- | --- |
| **C** | **D**  **** |
| **E** | **F** |

**Supplementary Figure 1.** Summary of cell filtering, hypoxia annotation, and cell type composition across experimental conditions.

**A)** Bar chart showing the number of cells retained after quality control filtering across control (CTRL), low-dose (LD), and high-dose (HD) samples for both 2D and 3D cultures. **B)** Bar chart displaying the number of cells annotated as hypoxic or normoxic in each condition. **C–D)** UMAP visualizations of all cells cultured in 2D and 3D, respectively, annotated as hypoxic (red) or normoxic (green) based on hypoxia marker expression. **E–F)** UMAP visualizations of 3D-cultured cells exposed to LD and HD APAP, respectively, annotated as hypoxic (red) or normoxic (green) based on hypoxia marker gene expression.

| **Gene** | **Hypoxia** | **Dose** | **Cell type** |
| --- | --- | --- | --- |
| HMOX1 | 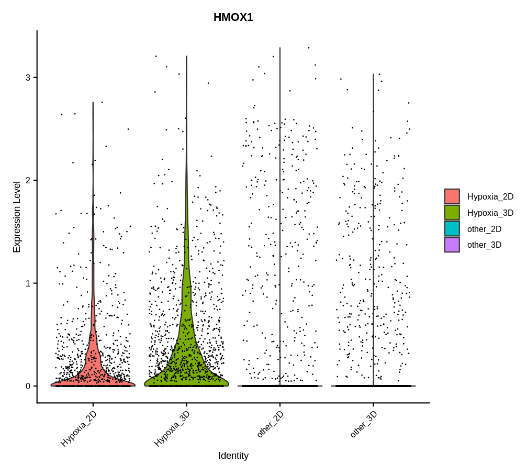 | 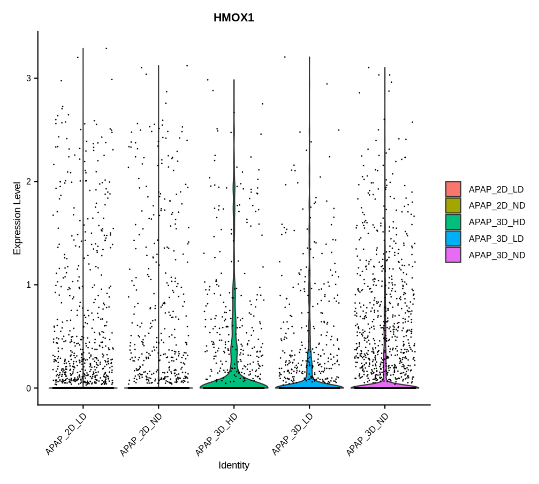 | 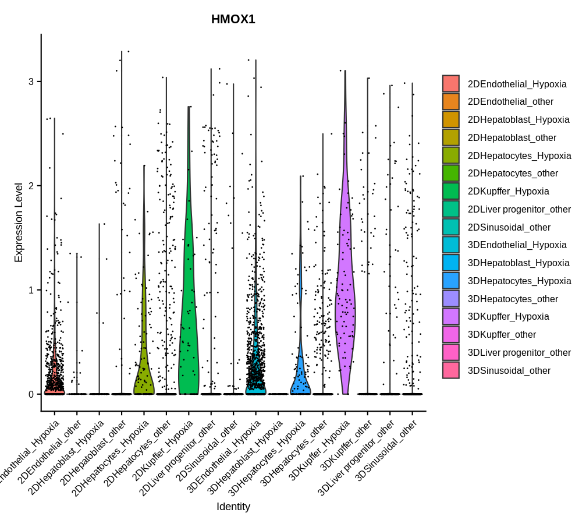 |
| GCLC | 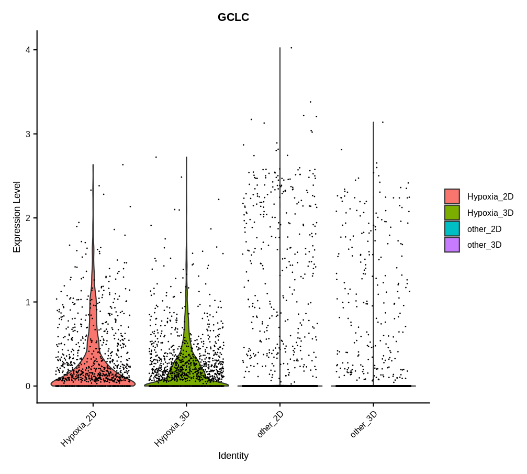 | 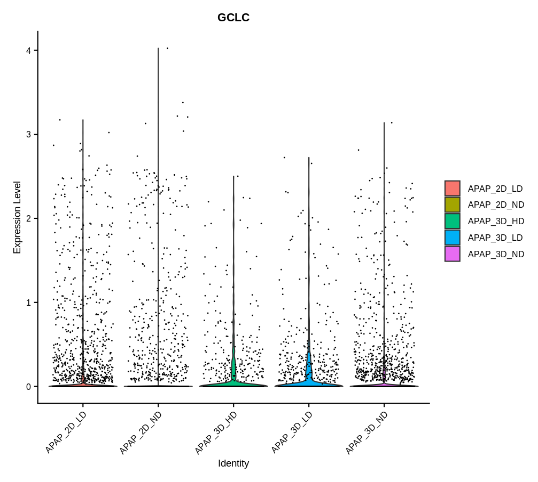 | 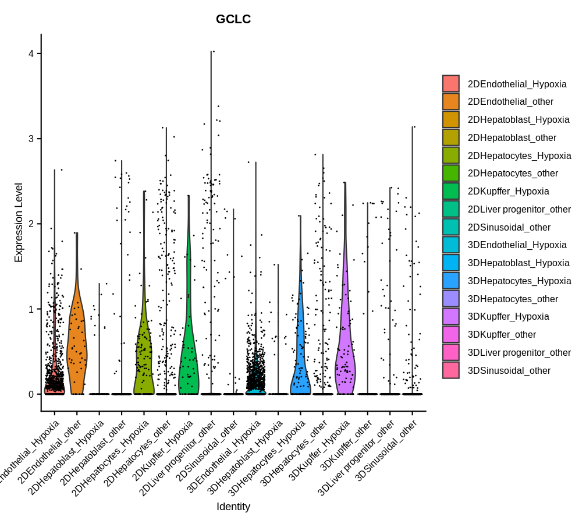 |
| NQO1 | 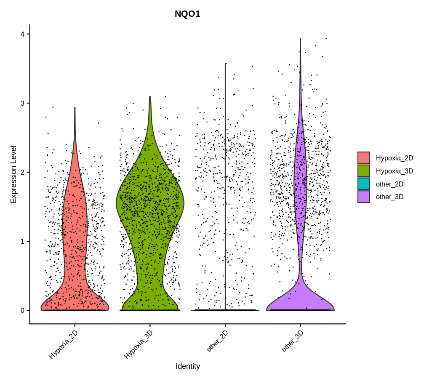 | 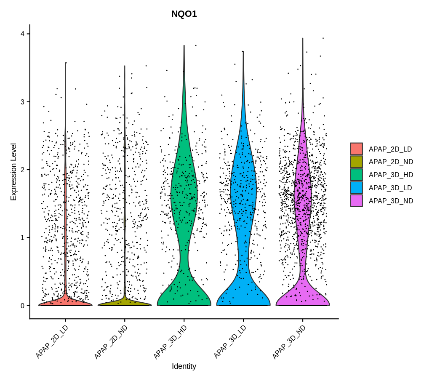 | 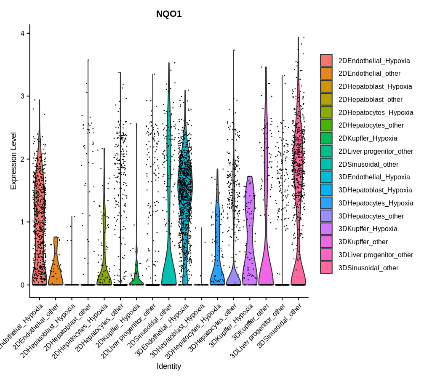 |
| JUN | 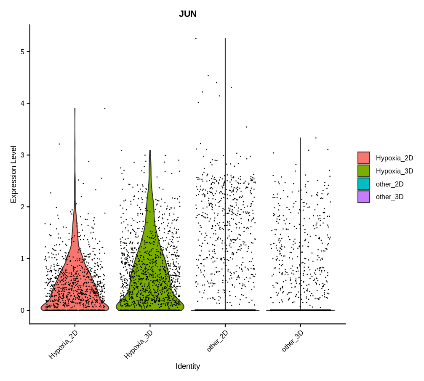 | 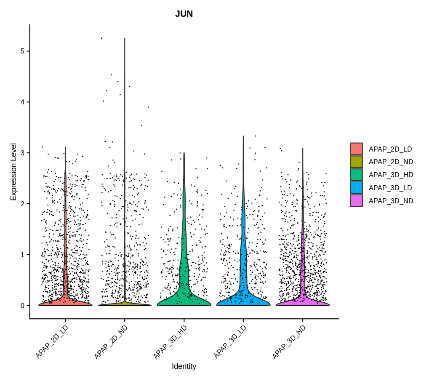 | 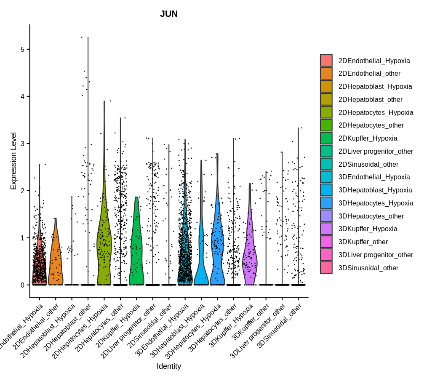 |
| MYC | 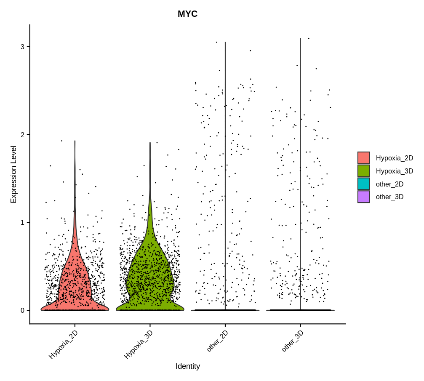 | 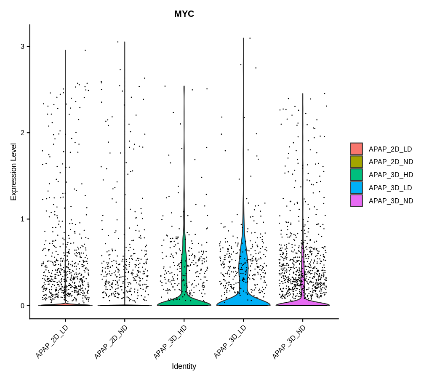 | 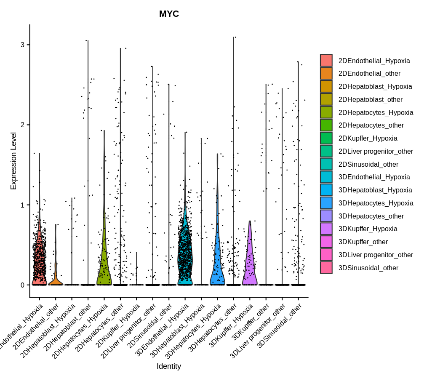 |
| DDIT3 | 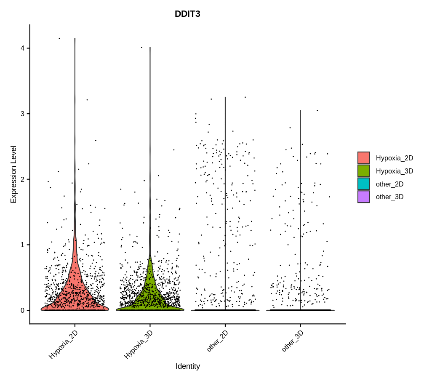 | 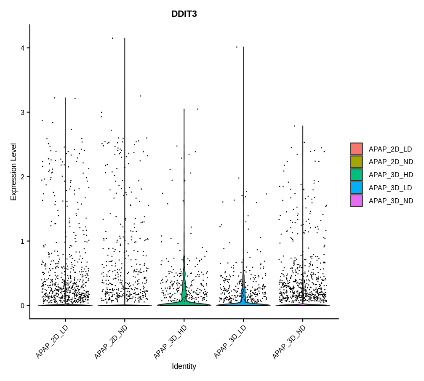 | 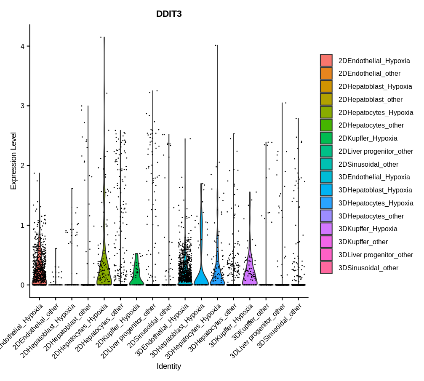 |
| GADD45A | 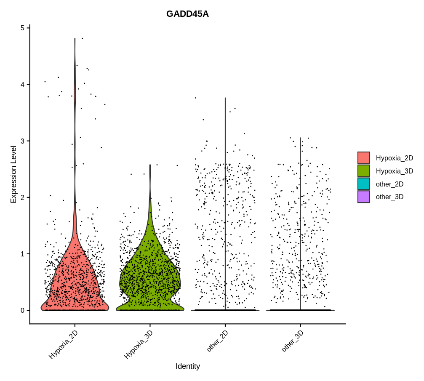 | 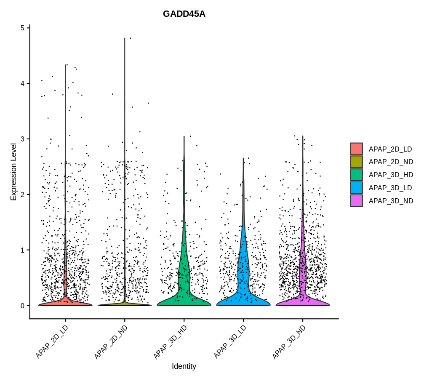 | 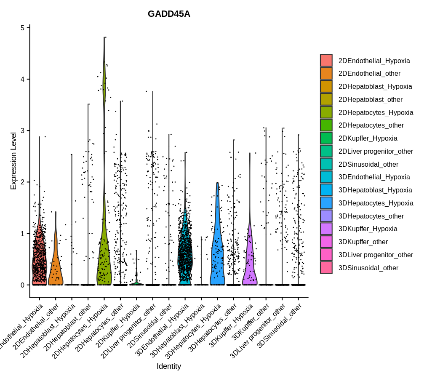 |
| GCLM | 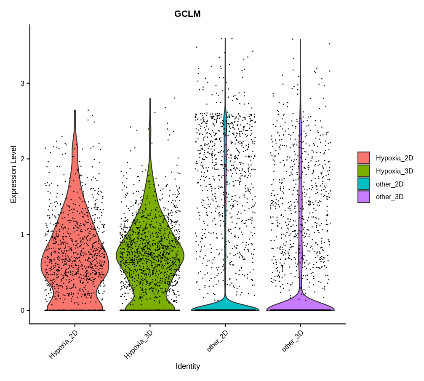 | 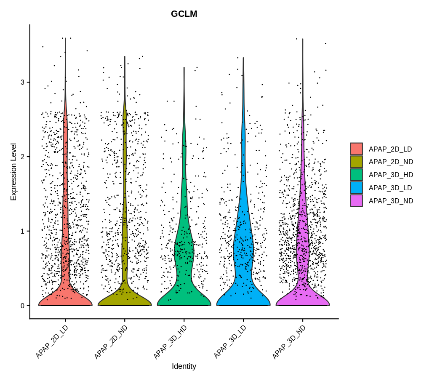 | 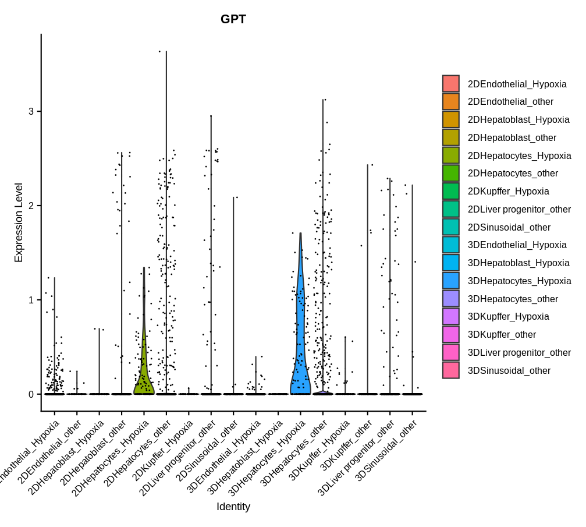 |
| CDKN1A | 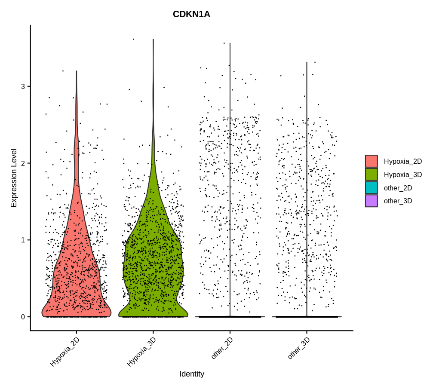 | 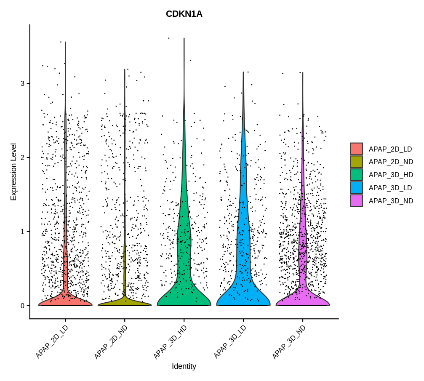 | 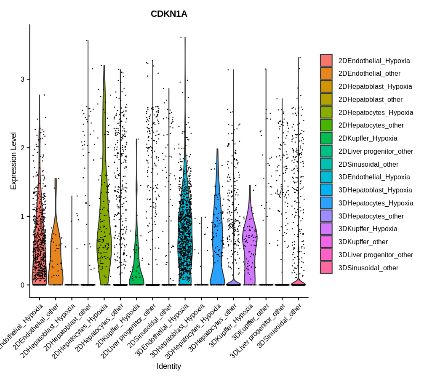 |
| EGR1 | 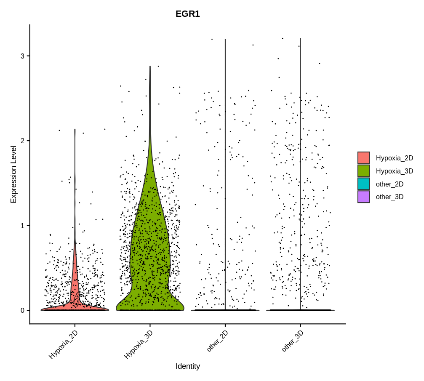 | 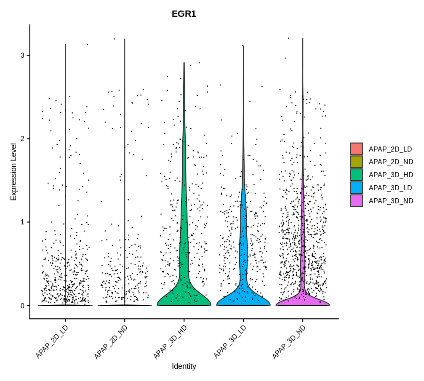 | 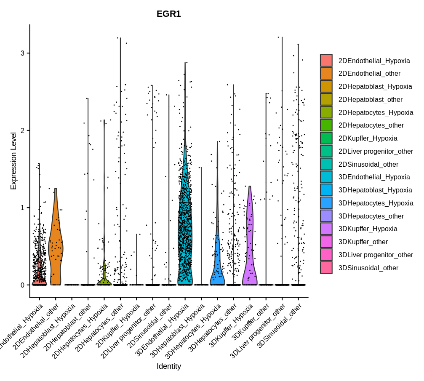 |
| FOS | 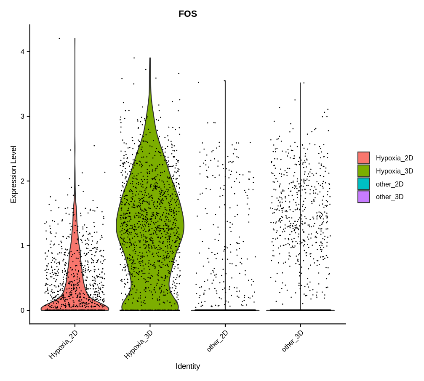 | 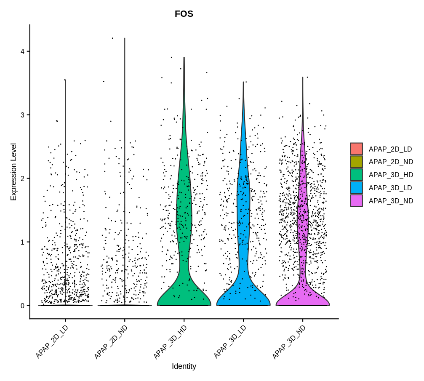 | 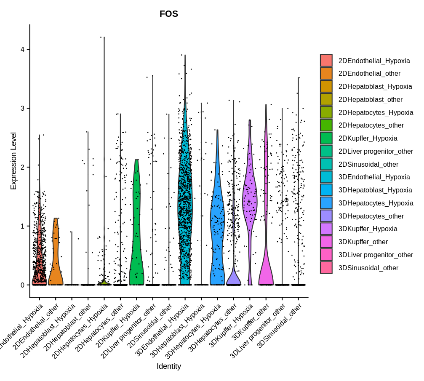 |
| SERPINE1 | 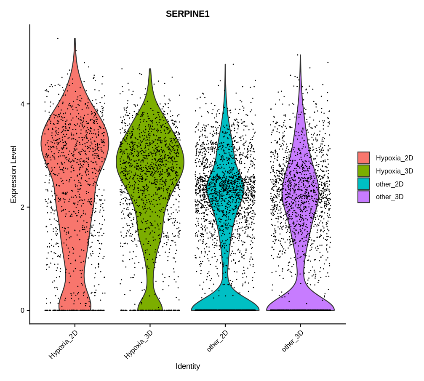 | 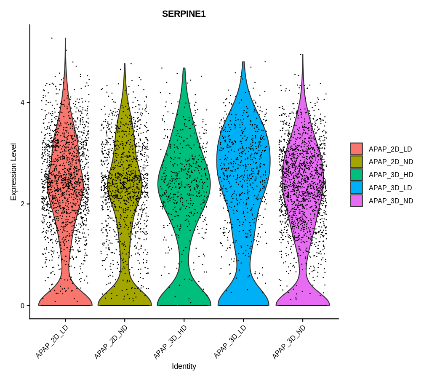 | 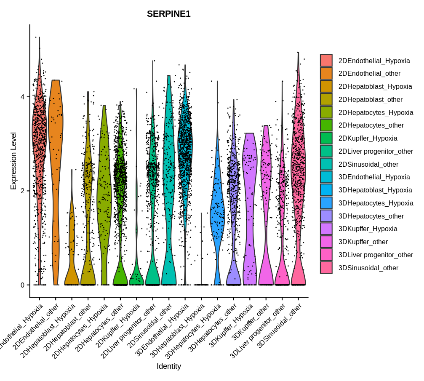 |
| BAX | 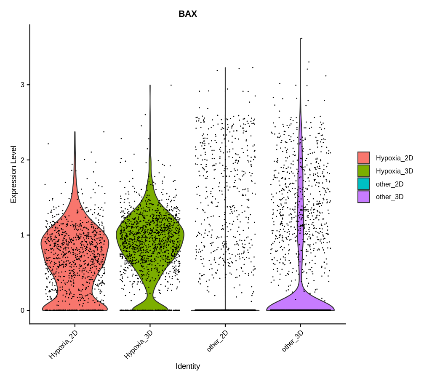 | 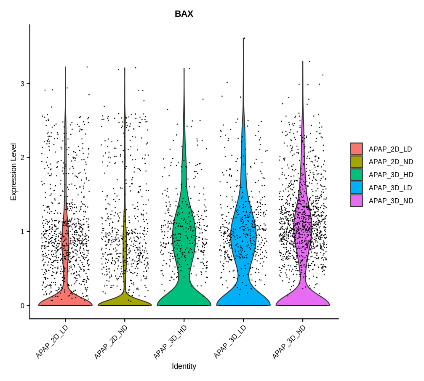 | 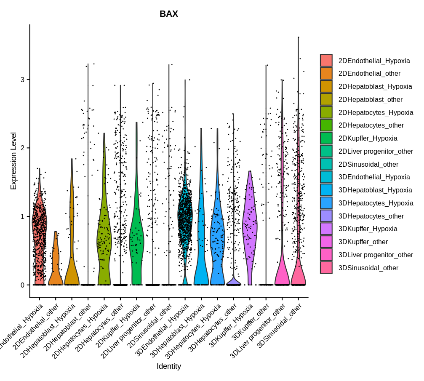 |
| DNAJB1 | 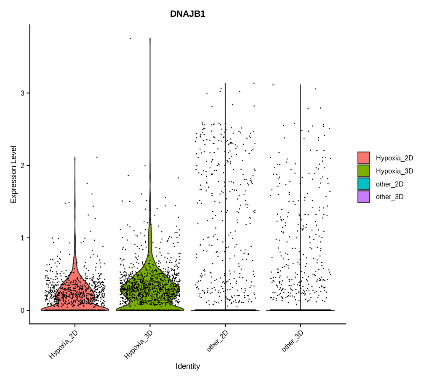 | 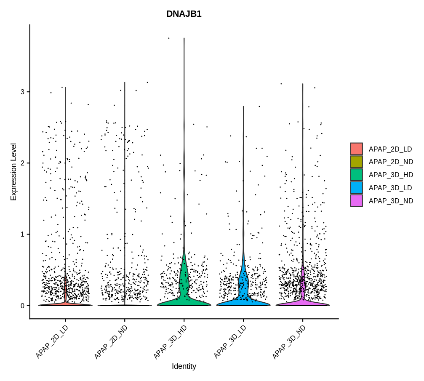 | 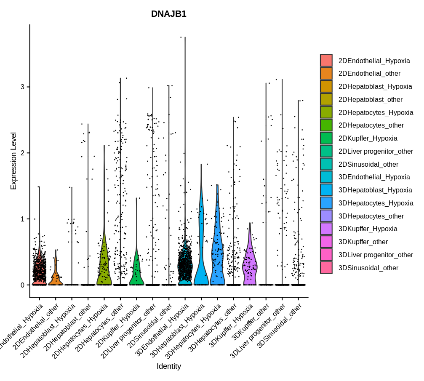 |

**Supplementary Figure 2.** Violin plots illustrating the expression levels of APAP-responsive genes (obtained from the Comparative Toxicogenomics Database) across different doses, culture conformations (2D and 3D), and hypoxia annotations. ND refers to the control group (no compound administered). “Other” denotes the normoxic cell population. Expression levels are further stratified by individual cell types, APAP exposure conditions, and hypoxia status

| A 2D Angiogenesis – ANGPTL4  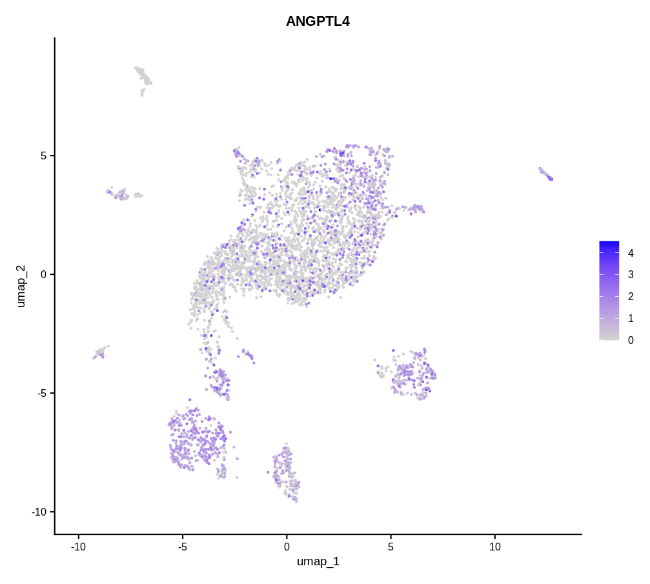 | B 3D Angiogenesis – ANGPTL4  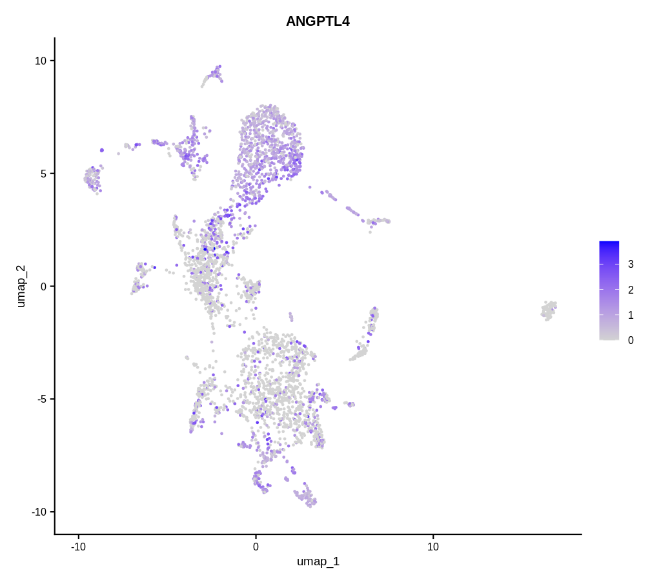 |
| --- | --- |
| C 2D Angiogenesis – VEGFA  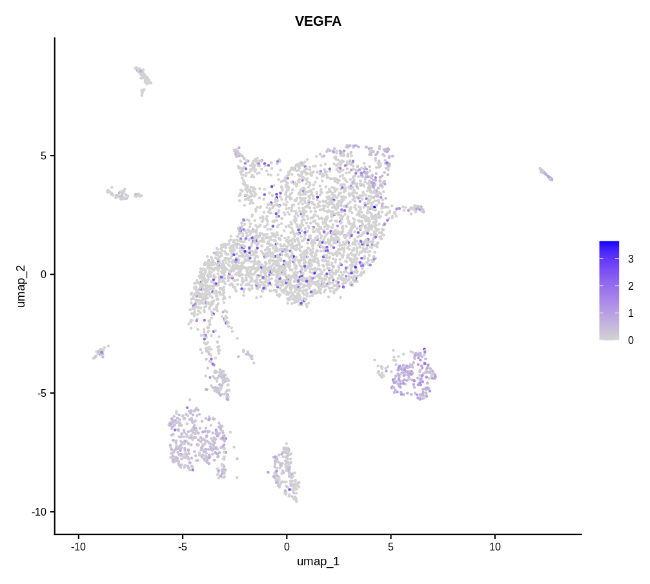 | D 3D Angiogenesis – VEGFA  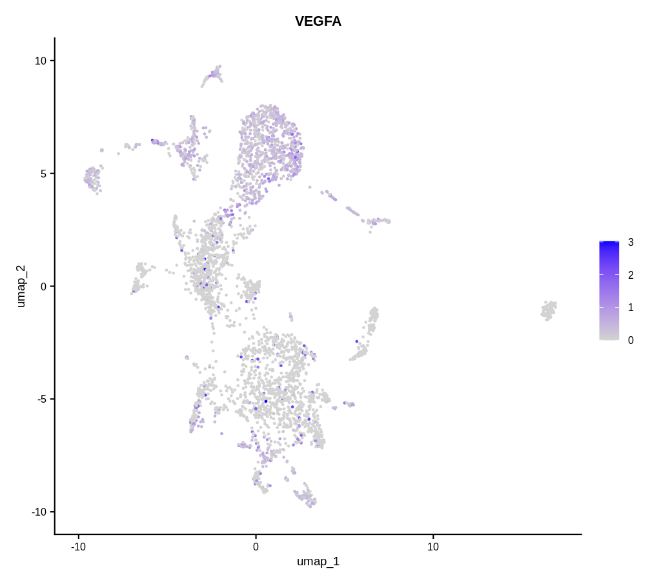 |
| E 2D Cell metabolism – LDHA  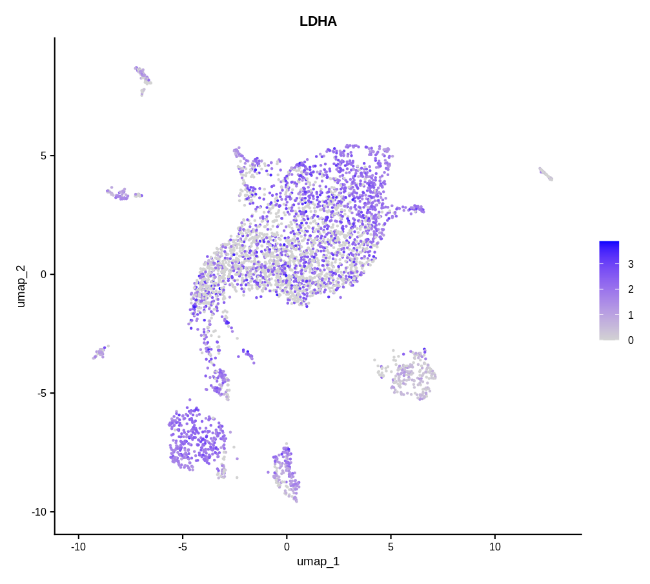 | F 3D Cell metabolism – LDHA  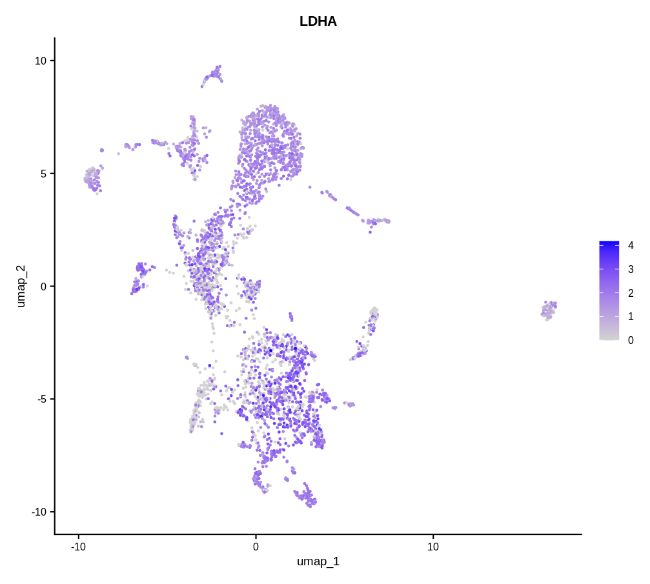 |
| G 2D Cell metabolism – SLC2A1  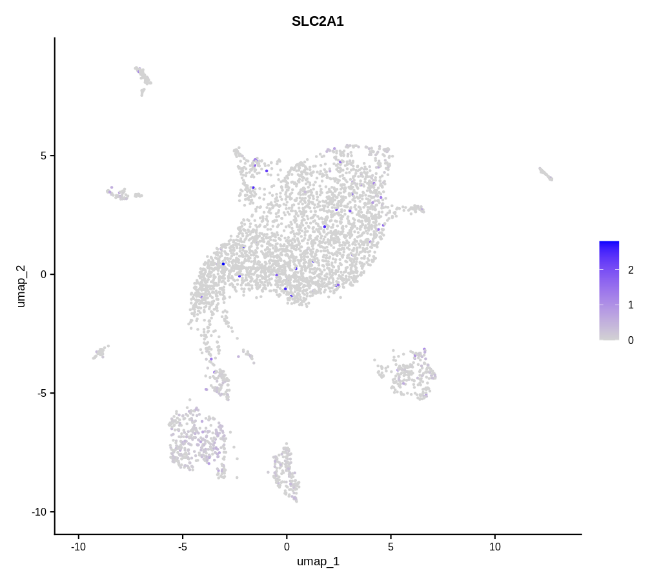 | H 3D Cell metabolism – SLC2A1  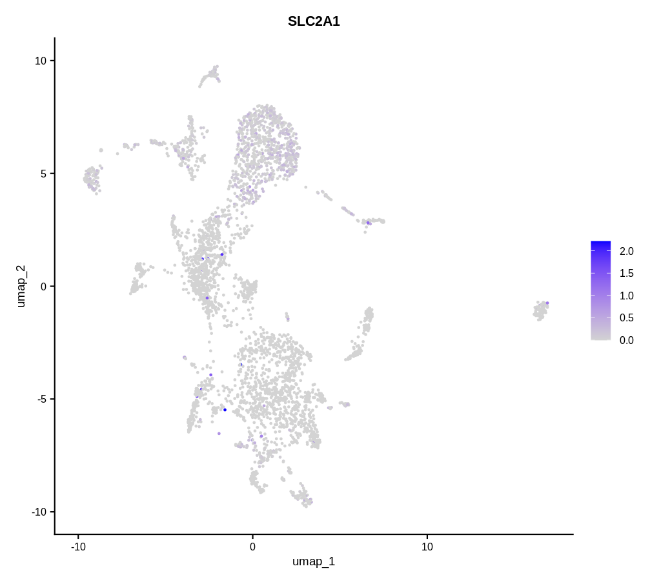 |
| I 2D epithelial-to-mesenchymal transition – LOX  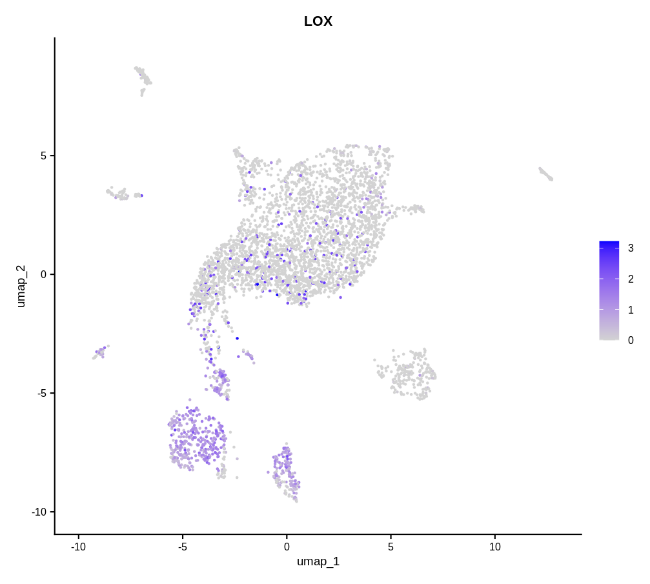 | J 3D epithelial-to-mesenchymal transition – LOX  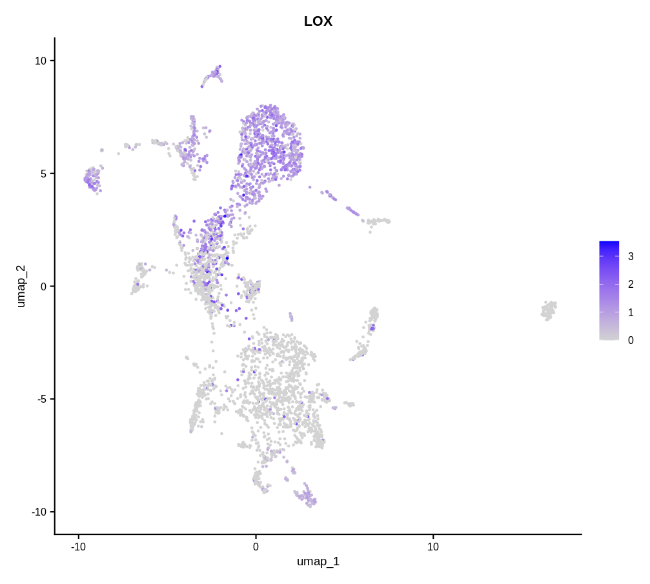 |
| K 2D epithelial-to-mesenchymal transition – P4HA1  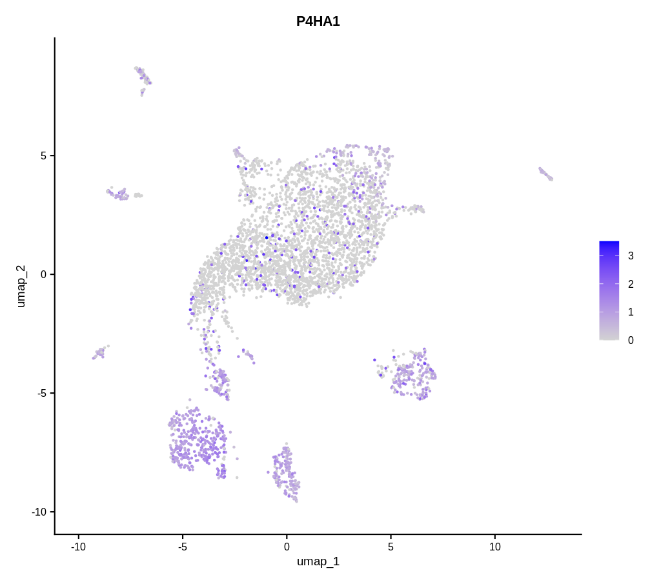 | L 3D epithelial-to-mesenchymal transition – P4HA1  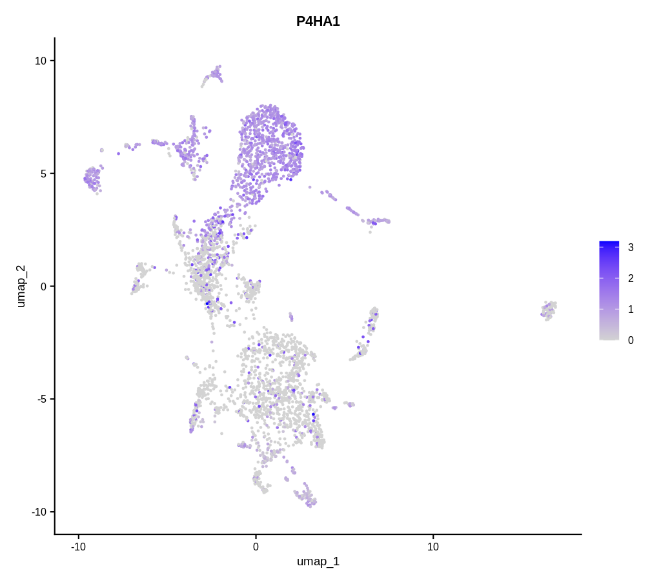 |
| M 2D Cell growth & survival – BNIP3  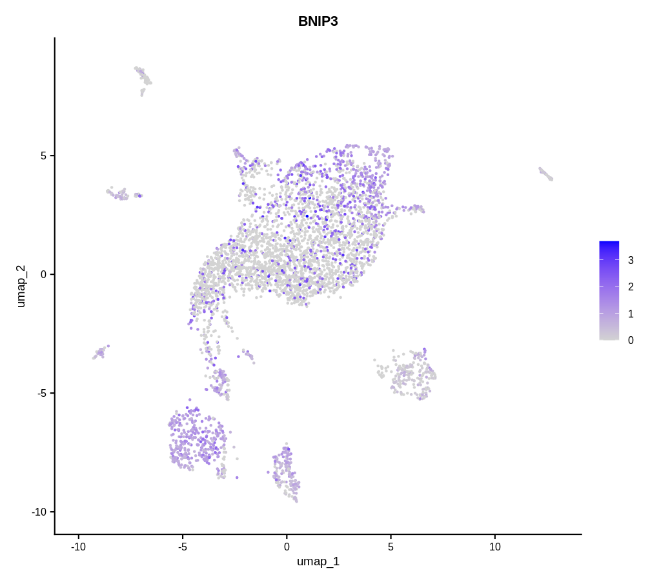 | N 3D Cell growth & survival – BNIP3  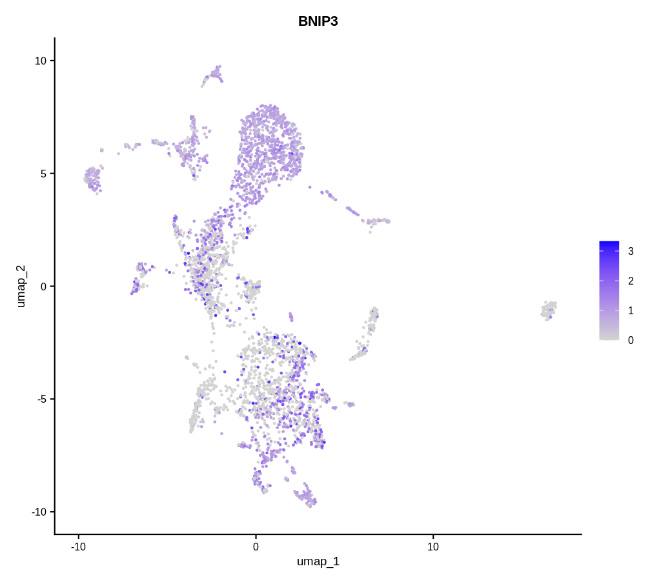 |
| O 2D Cell growth & survival – DDIT4  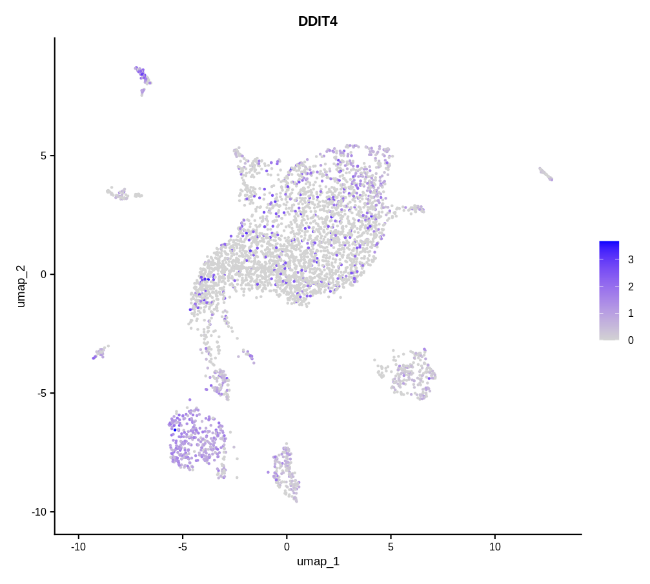 | P 3D Cell growth & survival – DDIT4  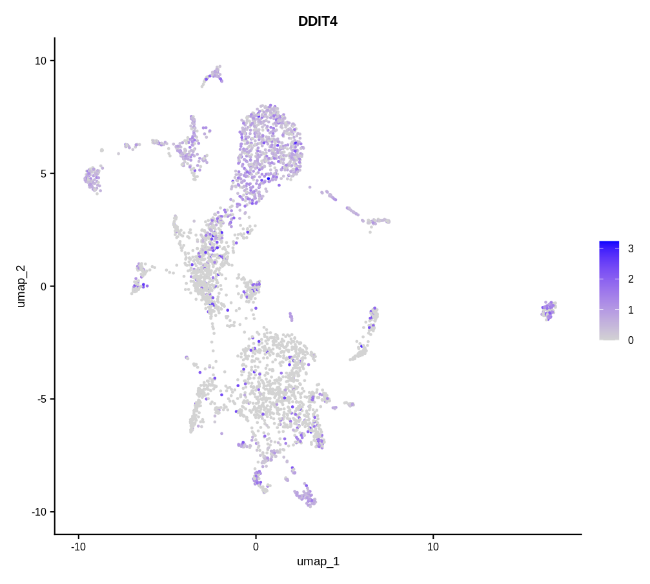 |

**Supplementary Figure 3.** UMAP visualizations showing expression levels of additional candidate hypoxia marker genes used for cell annotation in the 2D (left) and 3D (right) cell culture models.
